# Impact of Chinese visceral adiposity index on all-cause mortality risk in community-dwelling older adults: a prospective cohort study

**DOI:** 10.1007/s40520-024-02891-8

**Published:** 2024-12-03

**Authors:** Yuyu Zhang, Mingyue Shi, Zhao Dong, Tingting Li, Yangfan Gong, Wei Ge

**Affiliations:** Department of General Practice, Xijing Hospital, Fourth Military Medical University, Changle West Road #127, Xi’an, Shaanxi Province 710032 P.R. China

**Keywords:** Chinese visceral adiposity index, All-cause mortality, Older Chinese individuals, Cohort study

## Abstract

**Backgrounds:**

Whether excess visceral fat tissue increases the risk of death in older individuals remains controversial.

**Aims:**

To investigate the association between the Chinese Visceral Adiposity Index (CVAI) and all-cause mortality risk in older Chinese individuals.

**Methods:**

This cohort study utilized data of individuals aged ≥ 65 years in 2014 to 2018 wave from the Chinese Longitudinal Healthy Longevity Survey database. Older individuals in the 2014 wave were included and followed up in 2018. CVAI was calculated based on age, body size, and blood lipid parameters, with higher values indicating increased visceral fat. Survival status was determined from official death certificates, local primary healthcare providers, or the family members of participants. Kaplan-Meier survival curve and log-rank test were employed to analyze cumulative mortality risk through CVAI tertiles (tertile 1: CVAI index < 97.34; tertile 2: 97.43 ≤ CVAI index < 132.21; and tertile 3: CVAI index ≥ 132.21). A Cox proportional hazards regression model was used to assess the relationship between the CVAI groups and all-cause mortality risk. Additionally, a sensitivity analysis was performed by excluding participants who died within the first year of follow-up. A subgroup analysis was performed based on age and sex, and a restricted cubic spline plot was created to analyze the dose-response relationship between CVAI and mortality risk.

**Results:**

A total of 1414 individuals were included, and the mean age of the participants was 84.6 (standard deviation: 10.9) years, of which 46.4% were women and 32.8% were died during a median follow-up time of 36.4 months. In the multivariable adjusted Cox regression model, we observed a significantly lower risk of mortality in the CVAI tertile 2 and 3 groups than in the tertile 1 group. The hazard ratios (HR) of the tertile 2 and 3 groups were 0.68 (95% CI, approximately 0.52–0.89) and 0.63 (95% CI, approximately 0.48–0.82), respectively. Subgroup analysis revealed that the protective effect of higher CVAI levels on mortality was more pronounced in participants aged 65–79 years and in women.

**Conclusion:**

Our study established a linear relationship between CVAI and mortality risk among community-dwelling older adults, with higher CVAI levels associated with a lower risk of all-cause mortality. These findings highlight the potential importance of visceral adiposity in predicting mortality risk in community-dwelling older adults.

## Introduction

As the global population continues to age, exploring the risk factors threatening the health and well-being of older individuals has become important [1]. Obesity has been recognized as an important risk factor for multisystem diseases, particularly in older population for changes in body composition, which was characterized by reduced skeletal muscle mass and redistribution of visceral adiposity [2]. Although the limited data on the epidemiology of obesity in the elderly, the available studies suggested that the prevalence of obesity has increased in this population with more than 10% of the Chinese elderly population being classified as obese [3]. Body index mass (BMI) was the most commonly used index to assess obesity; however recent studies suggested that BMI cannot adequately evaluate the distribution of visceral adiposity and its related health risks in older adults [4, 5]. Techniques such as dual-energy X-ray absorptiometry, computed tomography, and magnetic resonance imaging have been used to evaluate the health risks associated with visceral fat [6, 7], but these methods were not suitable for community clinical practice.

The Chinese visceral adiposity index (CVAI), which incorporated age, BMI, waist circumference, and lipid metabolism parameters, was a specific indicator used to evaluate visceral adiposity and its impact on health outcomes [8, 9]. Studies have demonstrated that CVAI was a reliable biomarker for predicting the occurrence and prognosis of various diseases [10–13]. A recent study based on the China Health and Retirement Longitudinal Study database indicated that CVAI was associated with an increased risk of new-onset stroke, and this indicator was reliable and effective for stroke risk stratification [14]. Another study highlighted the correlation between CVAI and the development of proteinuria in community patients with diabetes and hypertension, suggesting that CVAI could be recognized as a key indicator in predicting albuminuria [15]. A large prospective study utilized the China HEART database to analyze the association between CVAI and cause-specific mortality, revealing that compared with individuals in the quantile 1 CVAI group, the risk of cardiovascular disease increased in the quantile 4 group [16]. However, due to changed physiological, metabolic, and lifestyle factors in older population, different health outcomes related to visceral adiposity might be established. Currently, the association between CVAI and all-cause mortality risk in older populations remains underexplored.

In this study, based on the Chinese Longitudinal Healthy Longevity Survey (CLHLS) database, we aimed to investigate the correlation between CVAI and all-cause mortality in older adults.

## Methods

### Data source and study participants

**Fig. 1:**
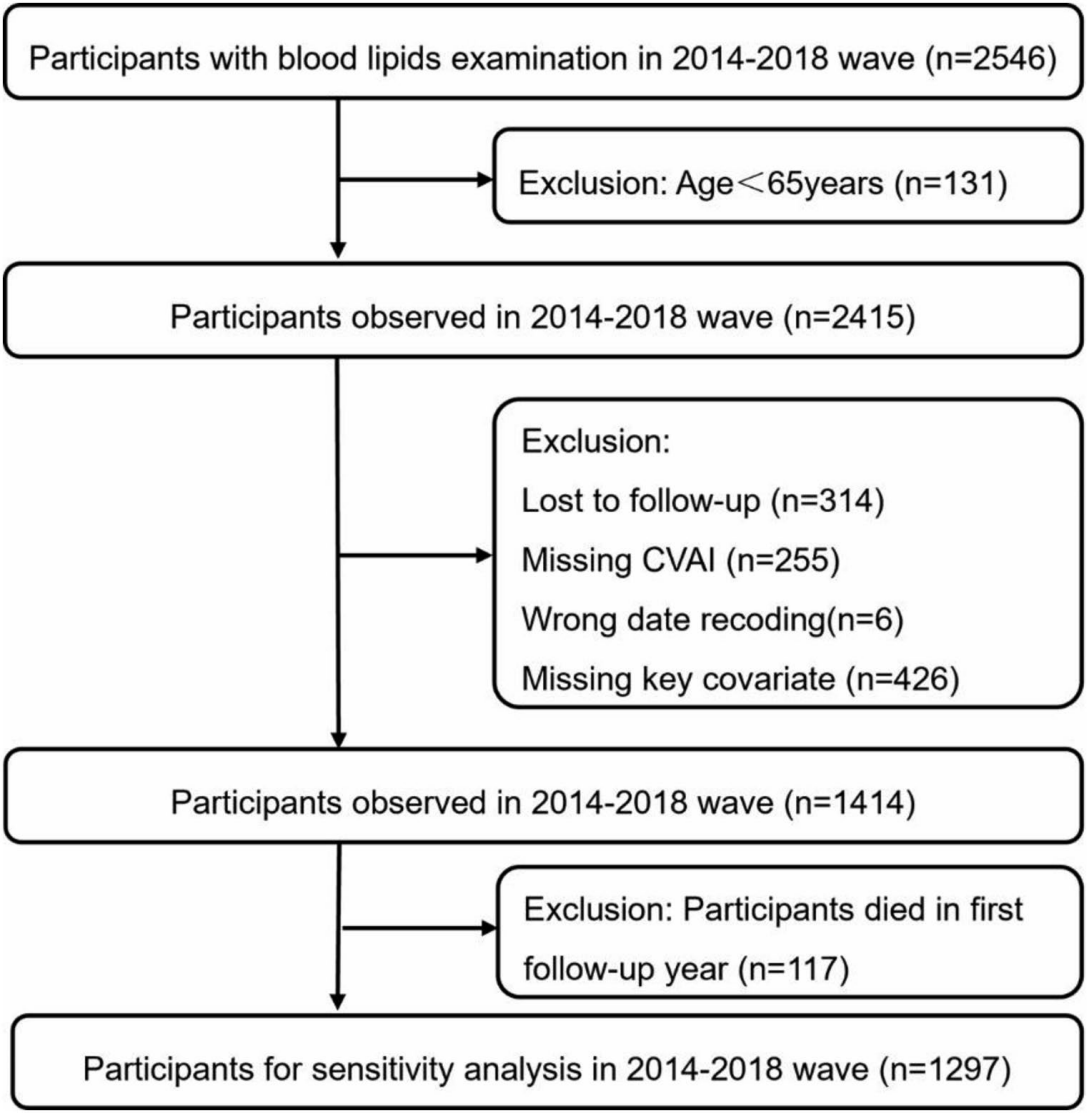
Flowchart of the selection of participants for blood lipid examination in the 2014–2018 wave

The data for this study were obtained from the CLHLS database, which is a representative dataset of the older population in China [17]. The dataset investigated the personal backgrounds, family structures, and lifestyles of individuals aged ≥ 65 years using a stratified multistage probability sampling method. The CLHLS survey was approved by the Biomedical Ethics Committee of Peking University and all participants provided informed consent. Researchers acquired the right to use the CLHLS data through the Peking University Open Research Data Platform, subsequently obtaining serum examination data from the 2014 wave and survey data from the 2014 to 2018 wave. Baseline data included the sociodemographic information, health-related behaviors, and lipid indicators of 2546 participants. The outcome was the survival status of the participants in the 2018 wave. After excluding individuals aged < 65 years, lacking lipid data, incorrect death dates, lost to follow-up, or lacking key covariates, 1414 participants were finally included. A flowchart of the participant selection is presented in Fig. 1.

### CVAI assessment

The CVAI, proposed in 2016, was more suitable for assessing visceral fat distribution in the Chinese population, and calculated using the following formula [8]:

Men: CVAI = − 267.93 + 0.68 ∗ age (years) + 0.03 ∗ BMI (kg/m^2^) + 4.00 ∗ WC (cm) + 22.00 ∗ Lg TG (mmol/L)– 16.32 ∗ HDL-C (mmol/L);

Women: CVAI = − 187.32 + 1.71 ∗ age (years) + 4.32 ∗ BMI (kg/m^2^) + 1.12 ∗ WC (cm) + 39.76 ∗ Lg TG (mmol/L)– 11.66 ∗ HDL-C (mmol/L).

CVAI was calculated based on age, body size, and blood lipid parameters. Age was obtained through self-reporting by participants or their family members. The body size parameters included height, weight, and waist circumference. These measurements were conducted by trained investigators following a standardized protocol. Participants were asked to stand upright with their feet shoulder-width apart and breathe calmly. Waist circumference was measured at the level of the navel using a tape measure, weight was recorded using an electronic scale, and height was measured with stadiometers. Each participant was examined twice and the mean value was recorded. For participants who were unable to stand, alternative methods were employed. Furthermore, BMI was calculated as body weight divided by height squared (kg/m^2^). Lipid parameters included triglyceride (TG) and high-density lipoprotein cholesterol (HDL-C) levels. Fasting blood samples were collected from local hospitals. A commercial diagnostic kit (Roche Diagnostics, Germany) was used to analyze the blood samples on an automated biochemistry machine (Hitachi 7180, Japan). The glycerol phosphate oxidase-peroxidase method was used to measure the TG and HDL-C levels. Higer CVAI indicates more visceral adipose tissue, and CVAI index was grouped by tertiles in this study (tertile 1: CVAI index < 97.34; tertile 2:97.43 ≤ CVAI index < 132.21; and tertile 3: CVAI index ≥ 132.21), with tertile 1 serving as the reference group.

### Outcome assessment

Survival status was acquired from official death certificates, local primary healthcare providers, or the family members of participants by trained investigators. The date of participation in the 2014 wave was regarded as the starting point, with the date of death for deceased individuals and the follow-up date in the 2014–2018 wave for surviving individuals as the endpoint. Survival time was measured for the entire observation period from 2014 to 2018.

Covariate variables comprising sociodemographic characteristics and health-related factors were included. Sociodemographic factors, including age, sex (men or women), residence (city/urban or rural), cohabitation with family (yes or no), and education (0 year or ≥ 1 years) were recorded using baseline questionnaires. Health-related behaviors were further included in the data analysis: current smoking status, current drinking status, self-reported quality of life, sleep quality, self-reported health, activities of daily living (ADL), instrumental ADL (IADL), eating fresh fruits, drinking tea, participating in social activities, and chronic disease scores. Self-reported quality of life was evaluated through a single-item question, asking them to assess their life quality across five categories: “very good,” “good,” “soso,” “bad,” and “very bad.” These responses were grouped into three categories: very good/good,” “soso,” and “bad/very bad.” Similarly, sleep quality (assessed by the question, How is the quality of your sleep? ) and self-reported health (assessed by the item How do you rate your health at present? ) were categorized as “very good/good,” “soso,” and “bad/very bad.” The ADL assessment reflected the self-care ability of each respondent, and IADL was an essential indicator for evaluating the daily function and independence of older individuals. One or more aspects of dependence were defined as ADL or IADL disability. The sum of five chronic diseases (ranging from 0 to 5), including hypertension, diabetes, heart disease, stroke/cerebrovascular diseases, and respiratory diseases (bronchitis, emphysema, pneumonia, and asthma), was measured as chronic disease scores in our analysis, which were categorized into three groups (no disease, one disease, and comorbidity).

### Statistical analysis

Continuous variables and the number of cases or percentages in categorical variables are described as mean ± standard deviation or median values (interquartile range), respectively. Student’s t-test or a non-parametric test was used for the comparison of continuous variables, and the chi-square test or Fisher’s exact probability method were used for categorical variables. Participants were stratified into three groups according to CVAI tertiles to explore the correlation between CVAI levels and mortality. Kaplan–Meier curves and log-rank tests were performed to compare the differences in survival probability among the CVAI tertiles. Cox proportional hazard regression was used to estimate hazard ratios (HR) and 95% confidence intervals (CI). We analyzed the association between the CVAI and all-cause mortality using unadjusted and adjusted models. The initial model (Model 1) was adjusted for sex, age, and residence. Based on Model 1, additional covariates, including current smoking status, current alcohol consumption, cohabitation with family, educational level, self-reported health, self-reported quality of life, and sleep quality, were adjusted for in Model 2. The remaining covariates, including eating fresh fruit, drinking tea, participation in social activities, ADL, IADL, and chronic disease scores, were further adjusted in Model 3. Subgroup analyses were performed to assess the consistency of the relationship between CVAI and all-cause mortality. Restricted cubic spline (RCS) analysis in a Cox proportional hazards model was performed to explore possible linear correlations between CVAI and mortality among older individuals. To clarify the robustness of our results, we performed a sensitivity analysis by excluding individuals who died during the first follow-up year.

Data processing and statistical analyses were performed using SPSS version 26.0 and R Statistical Software version 4.2.2. Statistical significance was set at *P* < 0.05. All tests were two-tailed.

## Results

### Baseline characteristics

The sociodemographic characteristics and health-related factors of the 1414 older individuals, stratified by survival status are presented in Table 1. The mean age of the participants included in our study was 84.6 (standard deviation: 10.9) years, of which 950 (67.2%) were still alive, and 46.4% were women. Compared to participants who were still alive at the follow-up date, those who had died were more likely to be older and women. The mean CVAI value was 114.9 ± 39.8, and was higher in the participants who had died. A statistically significant difference was observed in the CVAI groups. Male sex, being married, current smokers, current drinkers, higher self-reported quality of life, and self-reported health were more common among the survivors. Participants who were alive were more likely to consume fresh fruit, drink tea, and engage in social activities. The percentage of patients with at least one ADL or IADL limitation at death was 25.2% and 80.4%, respectively, which was higher in the survival group. No significant differences were observed in residence, living with family, sleep quality, or chronic disease scores.

**Table 1:** Baseline characteristics of participant

Variables	Total (*n* = 1414)	Survival (*n* = 950)	Death (*n* = 464)	*P* value
Age at baseline, Mean ± SD, years	84.6 ± 10.9	80.9 ± 9.7	92.2 ± 9.2	< 0.001
Categories, n (%)				< 0.001
65-79years	490 (34.7)	449 (47.3)	41 (8.8)	
80-89years	442 (31.3)	311 (32.7)	131 (28.2)	
>90years	482 (34.1)	190 (20)	292 (62.9)	
Sex, n (%)				< 0.001
Male	694 (49.1)	507 (53.4)	187 (40.3)	
Female	720 (50.9)	443 (46.6)	277 (59.7)	
Residence, n (%)				0.831
City/urban	294 (20.8)	196 (20.6)	98 (21.1)	
Rural	1120 (79.2)	754 (79.4)	366 (78.9)	
Education				< 0.001
0	860 (60.8)	511 (53.8)	349 (75.2)	
≥ 1	554 (39.2)	439 (46.2)	115 (24.8)	
Marital status				< 0.001
Married	618 (43.7)	506 (53.3)	112 (24.1)	
Divorced/widowed/ never married	796 (56.3)	444 (46.7)	352 (75.9)	
Current smoking, n (%)				0.001
Yes	219 (15.5)	168 (17.7)	51 (11)	
No	1195 (84.5)	782 (82.3)	413 (89)	
Current drinking, n (%)				0.009
Yes	218 (15.4)	163 (17.2)	55 (11.9)	
No	1196 (84.6)	787 (82.8)	409 (88.1)	
Living with family, n (%)				0.999
Yes	1094 (77.4)	735 (77.4)	359 (77.4)	
No	320 (22.6)	215 (22.6)	105 (22.6)	
Self-reported life quality, n (%)				0.023
Good/very good	956 (67.6)	658 (69.3)	298 (64.2)	
Soso	423 (29.9)	275 (28.9)	148 (31.9)	
Bad/very bad	35 (2.5)	17 (1.8)	18 (3.9)	
Self-reported health, n (%)				< 0.001
Good/very good	757 (53.5)	538 (56.6)	219 (47.2)	
Soso	524 (37.1)	350 (36.8)	174 (37.5)	
Bad/very bad	133 (9.4)	62 (6.5)	71 (15.3)	
Sleep quality, n (%)				0.888
Good/very good	961 (68.0)	648 (68.2)	313 (67.5)	
Soso	349 (24.7)	231 (24.3)	118 (25.4)	
Bad/very bad	104 (7.4)	71 (7.5)	33 (7.1)	
Eat fruit, n (%)				< 0.001
Everyday	675 (47.7)	487 (51.3)	188 (40.5)	
Sometimes or occasionally	486 (34.4)	317 (33.4)	169 (36.4)	
Never	253 (17.9)	146 (15.4)	107 (23.1)	
Drinking tea, n (%)				0.022
Yes	501 (35.4)	356 (37.5)	145 (31.2)	
No	913 (64.6)	594 (62.5)	319 (68.8)	
Participant in social activity, n (%)				< 0.001
Yes	120 (8.5)	101 (10.6)	19 (4.1)	
No	1294 (91.5)	849 (89.4)	445 (95.9)	
ADL, n (%)				< 0.001
Normal	1255 (88.8)	908 (95.6)	347 (74.8)	
Abnormal	159 (11.2)	42 (4.4)	117 (25.2)	
IADL, n (%)				< 0.001
Normal	665 (47.0)	574 (60.4)	91 (19.6)	
Abnormal	749 (53.0)	376 (39.6)	373 (80.4)	
Chronic disease scores, n (%)				0.158
No	804 (56.9)	552 (58.1)	252 (54.3)	
One disease	421 (29.8)	282 (29.7)	139 (30)	
Comorbidity	189 (13.4)	116 (12.2)	73 (15.7)	
CVAI, Mean ± SD	114.9 ± 39.8	112.6 ± 40.5	119.7 ± 38.0	0.002
CVAI, n (%)				0.003
Tertile 1 (<97.34)	471 (33.3)	337 (35.5)	134 (28.9)	
Tertile 2 (97.43-132.21)	471 (33.3)	323 (34)	148 (31.9)	
Tertile 3 (>132.21)	472 (33.4)	290 (30.5)	182 (39.2)	

*Abbreviations* ADL, Activity of Daily Living; IADL, Instrumental Activity of Daily Living; CVAI, Chinese visceral adiposity index

### Association between CVAI and all-cause mortality risk

**Fig. 2:**
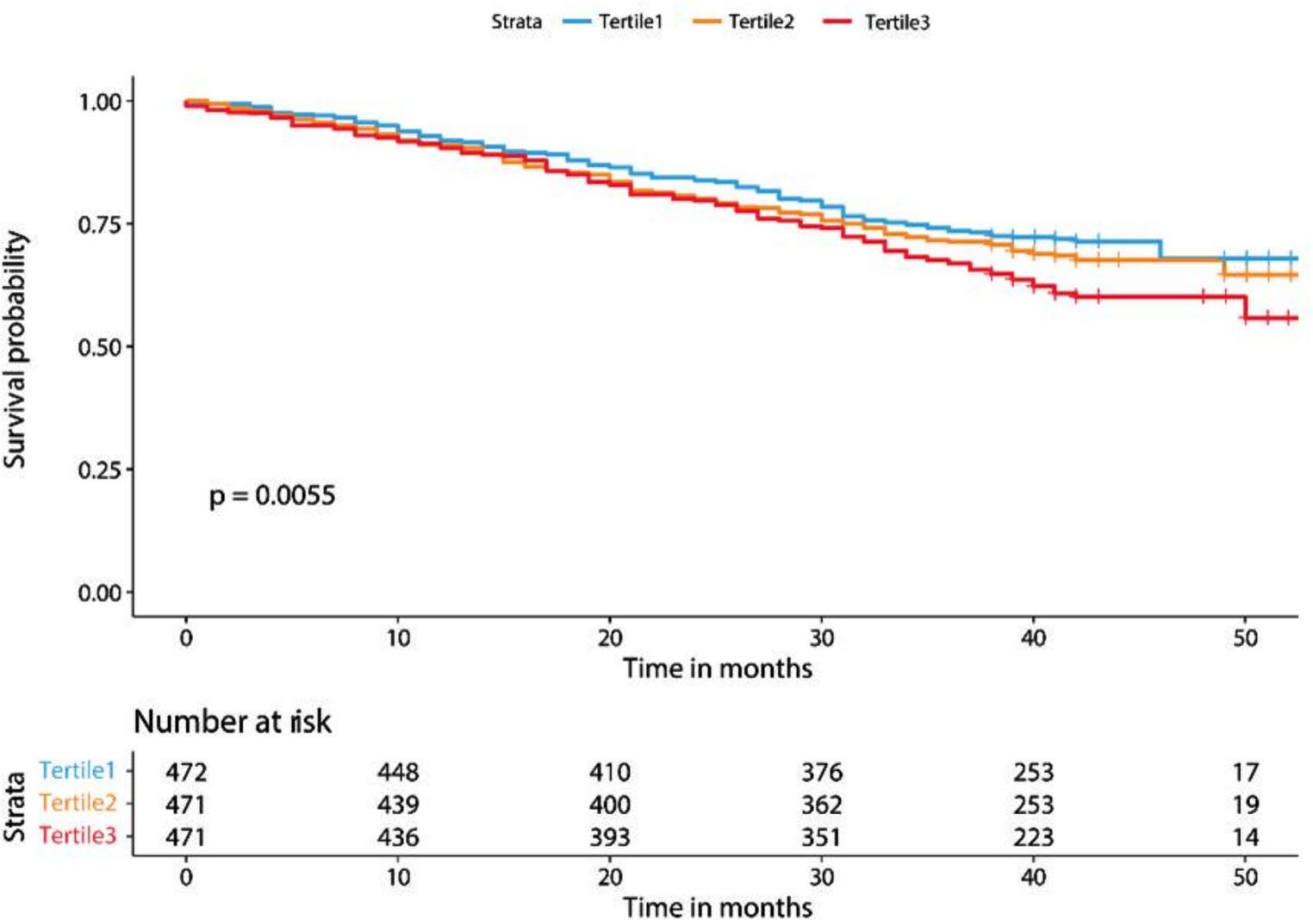
Kaplan–Meier survival curve and survival probability difference of the Chinese Visceral Adiposity Index (CVAI) tertile groups

**Fig. 3:**
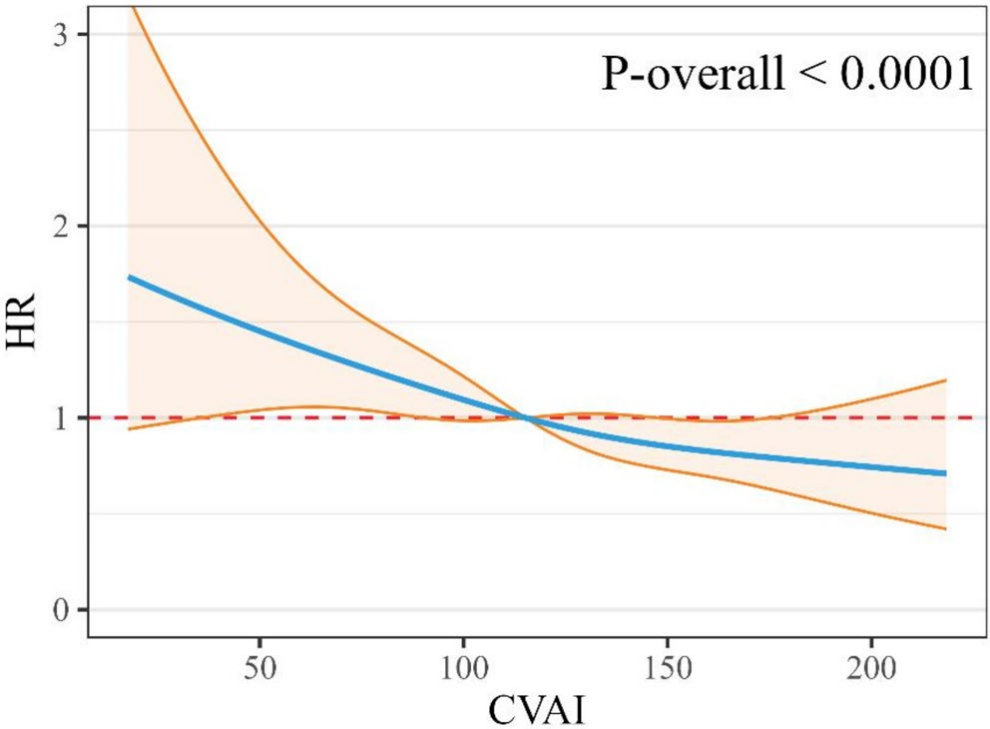
A restricted cubic spline (RCS) function was used to analyze the nonlinear relationship between the CVAI and all-cause mortality risk in older Chinese individuals using a Cox proportional hazards model (Model 3: adjusted for adjusted for sex, age, residence current smoking status, current alcohol consumption, cohabitation with family, educational level, self-reported health, self-reported quality of life, and sleep quality, eating fresh fruit, drinking tea, participation in social activities, ADL, IADL, and chronic disease scores)

During a median follow-up of 36.4 months, 462 (32.8%) of the 1414 participants died. The proportional hazard assumption was met for survival analysis using the Schoenfeld residual plot method. Figure 2 presents the Kaplan–Meier survival curves and log-rank test, which showed a significant difference between the CVAI tertiles (*P* = 0.0055). Table 2 shows the association between baseline CVAI and mortality risk with unadjusted and adjusted HRs and 95% CIs. In the unadjusted analysis, individuals in the tertile 3 (HR = 1.43, 95% CI = approximately 1.15–1.79) CVAI group had significantly higher hazard rates than those in the tertile 1 group, whereas the tertile 2 (HR = 1.13, 95% CI = approximately 0.9–1.43) group, no statistical differences were observed. In the multivariable adjusted model, compared with the tertile 1 group, participants in the tertile 2 and 3 groups had significantly lower risk of mortality with an HR of 0.68 (95% CI, approximately 0.52–0.89) and 0.63 (95% CI, approximately 0.48–0.82), respectively. And there was a significant trend of decreasing hazard of risk of mortality with higher CVAI level (HR = 0.81, 95% CI = approximately 0.7–0.92). The results of the sensitivity analyses are presented in Table 3. After excluding participants who died in first follow-up year, 1297 individuals were analyzed and the reduced risk was also observed in the tertile 2 (HR = 0.69, 95% CI = approximately 0.51–0.94) and 3 (HR = 0.67, 95% CI = approximately 0.49–0.92) groups. Figure 3 shows the smoothed spline curve of the HR for CVAI as a continuous variable in the full adjustment model. A linear relationship was observed between the CVAI levels and mortality (*P* < 0.001 for overall), and the risk of mortality decreased with increasing CVAI levels.

**Table 2:** Association between CVAI and risk of all-cause mortality

Categories	No.event_%	Crude	Model 1	Model 2	Model 3
		HR (95%CI)	HR (95%CI)	HR (95%CI)	HR (95%CI)
Tertile1	134 (28.5)	1(Ref)	1(Ref)	1(Ref)	1(Ref)
Tertile2	148 (31.4)	1.13 (0.9 ~ 1.43)	0.72 (0.56 ~ 0.93)*	0.68 (0.53 ~ 0.89)*	0.68 (0.52 ~ 0.89)*
Tertile3	182 (38.6)	1.43 (1.15 ~ 1.79)	0.73 (0.56 ~ 0.95)*	0.69 (0.53 ~ 0.91)*	0.63 (0.48 ~ 0.82)*
HR for tertile trend	464 (32.8)	1.2 (1.07 ~ 1.34)	0.87 (0.76 ~ 0.99)*	0.85 (0.74 ~ 0.97)*	0.81 (0.7 ~ 0.92)*

Note: *, *P*<0.05. Model 1: adjusted for sex, age, and residence; Model 2: adjusted for sex, age, residence current smoking status, current alcohol consumption, cohabitation with family, educational level, self-reported health, self-reported quality of life, and sleep quality; Model 3: adjusted for adjusted for sex, age, residence current smoking status, current alcohol consumption, cohabitation with family, educational level, self-reported health, self-reported quality of life, and sleep quality, eating fresh fruit, drinking tea, participation in social activities, ADL, IADL, and chronic disease scores

**Table 3:** Sensitive analysis

Categories	No.event_%	Crude	Model 1	Model 2	Model 3
		HR (95%CI)	HR (95%CI)	HR (95%CI)	HR (95%CI)
Tertile1	100 (22.9)	1(Ref)	1(Ref)	1(Ref)	1(Ref)
Tertile2	106 (24.7)	0.69 (0.51 ~ 0.94)*	0.71 (0.53 ~ 0.96)*	0.69 (0.51 ~ 0.94)*	0.69 (0.51 ~ 0.94)*
Tertile3	141 (32.7)	0.67 (0.49 ~ 0.92)*	0.77 (0.57 ~ 1.05)	0.76 (0.56 ~ 1.04)	0.67 (0.49 ~ 0.92)*
HR for tertile trend	347 (26.8)	0.84 (0.71 ~ 0.98)*	0.9 (0.77 ~ 1.05)	0.9 (0.77 ~ 1.05)	0.84 (0.71 ~ 0.98)*

Note: *, *P*<0.05. Model 1: adjusted for sex, age, and residence; Model 2: adjusted for sex, age, residence current smoking status, current alcohol consumption, cohabitation with family, educational level, self-reported health, self-reported quality of life, and sleep quality; Model 3: adjusted for adjusted for sex, age, residence current smoking status, current alcohol consumption, cohabitation with family, educational level, self-reported health, self-reported quality of life, and sleep quality, eating fresh fruit, drinking tea, participation in social activities, ADL, IADL, and chronic disease scores

### Subgroup analysis

**Fig. 4:**
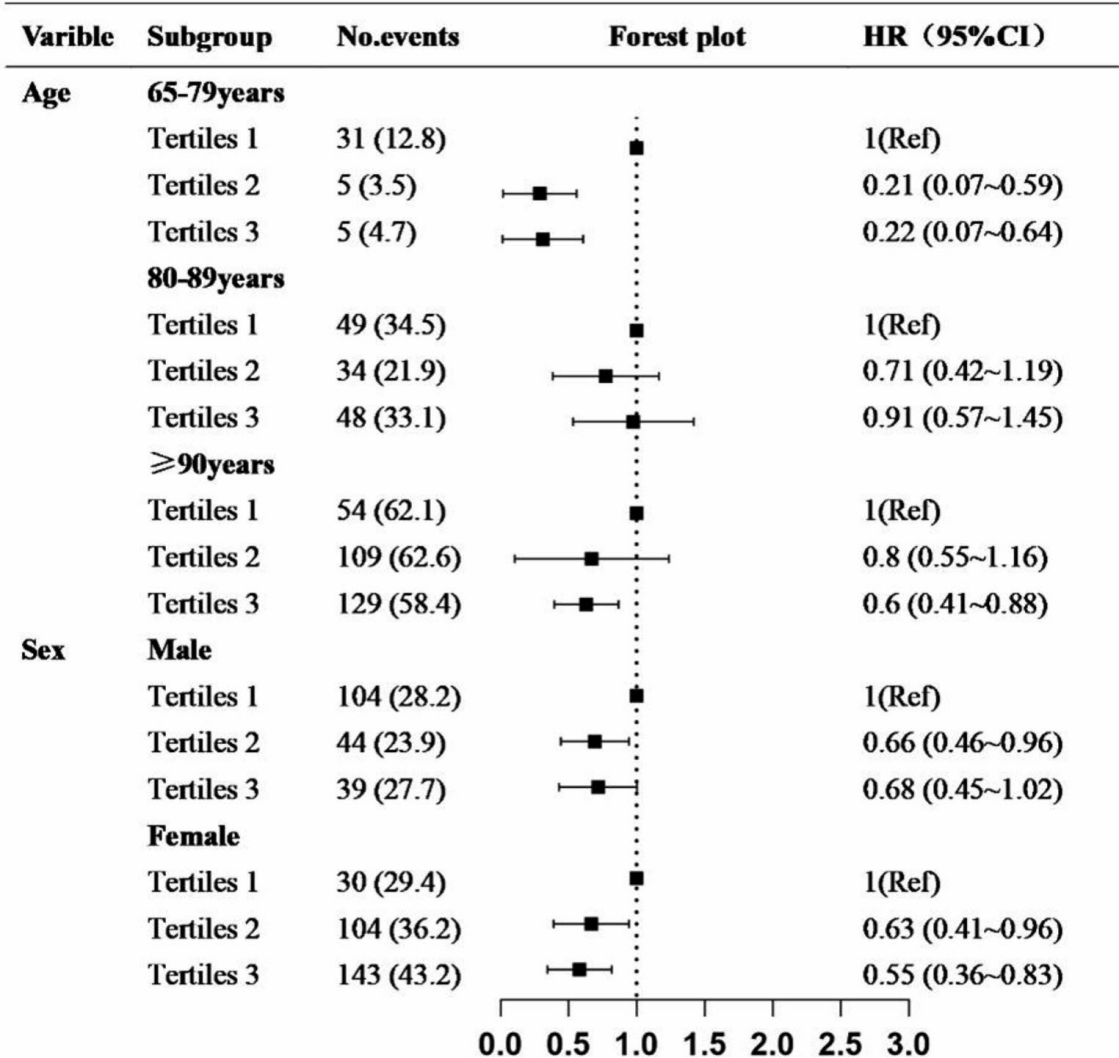
Subgroup analysis of the stratified results for different ages and sexes in a Cox proportional hazards model (Model 3: adjusted for adjusted for sex, age, residence current smoking status, current alcohol consumption, cohabitation with family, educational level, self-reported health, self-reported quality of life, and sleep quality, eating fresh fruit, drinking tea, participation in social activities, ADL, IADL, and chronic disease scores)

Figure 4 presents the stratified results for different age groups and sexes. Notably, among individuals aged < 79 years, the risk of mortality was reduced in participants corresponding to a 79% reduced mortality risk in the CVAI tertile 2 group (HR: 0.21, 95% CI: 0.07–0.59) and 78% in the tertile 3 group (HR: 0.22, 95% CI: 0.07–0.64). Conversely, no substantial differences were observed among participants aged 80–89 years or those aged > 90 years, even after the adjustments. Furthermore, the CVAI values were associated with a decreased risk of mortality in women in the tertile 2 (HR: 0.63, 95% CI: 0.41–0.96) and tertile 3 (HR: 0.55, 95% CI: 0.36–0.83) groups.

## Discussion

Our study found that a higher CVAI may reduce the risk of all-cause mortality among community-dwelling older adults. Compared with the CVAI tertile 1 group, the mortality risk was reduced by 32% and 37% in the tertile 2 and 3 groups, respectively. After excluding participants who died within the first follow-up year, the mortality risk in the tertile 2 and 3 groups were still significantly decreased. This protective effect was more pronounced in participants aged 65–79 years and in women. Our results also indicated that older individuals with lower CVAI levels had the highest risk of mortality.

The CVAI, a quantitative method to assess visceral adipose distribution in the Chinese population, refers to the evaluation of abnormal fat deposition around organs and tissues such as the liver, heart, and skeletal muscle [8, 18]. Currently, no consensus exists on the association between visceral adipose tissue accumulation and mortality risk in older individuals. Most studies have shown that visceral adipose tissue accumulation was positively associated with mortality in older individuals. A study conducted in Brazil revealed that older individuals experienced a 40% increase in all-cause mortality with increased visceral adipose tissue [19]. Another study indicated that the risk of all-cause mortality increased by 22% after adjusting for confounding factors, but no association was observed after adjusting for all confounders [20]. However, some studies, including ours, have revealed the protective effects of excessive visceral adipose tissue on mortality rates in older individuals. A study involving 1000 older individuals analyzed the association between quantified visceral fat tissue and all-cause mortality risk, and showed a 36% decrease in all-cause mortality rates [21]. Another investigation examining the association between obesity indicators in older individuals and mortality risk found a 14% decrease in mortality rates among older men [22]. A longitudinal study revealed that being overweight or mildly obese in individuals aged ≥ 80 years may be beneficial for health longevity [23]. Prospective studies focusing on older populations and mortality rates have demonstrated that excessive visceral adiposity does not increase the risk of all-cause mortality [24, 25].

Our study findings indicated a linear association between CVAI and mortality risk in the older population. Higher CVAI levels were associated with decreased mortality risk, suggesting that higher CVAI levels serve as a protective factor in older individuals. However, a recent cohort study highlighted a U-shaped relationship [16]. These discrepancies could be attributed to various factors, including differences in the age and sex distributions of the study participants. In our study, the average age of the individuals was 84.6 years, whereas the previous study focused on an younger population, with an average age of 57 years. To account for the potential differences in outcomes caused by age and sex, we conducted a subgroup analysis. The analysis showed that the reduced mortality risk associated with CVAI was particularly significant in individuals aged 65–79 years and in women. Possible explanations are as follows: the function of various organs and systems declines with increasing age; however, lifestyles and health management awareness may still be relatively good, which contributes to reducing the mortality risk in this age group. Biological parameters and lifestyle factors (such as inflammation status, endocrine function, and physical activity frequency) may affect the hormone levels of older women [26], leading to differences in visceral adipose tissue distribution between different sex subgroups [27]. Furthermore, participants in this age group may have more frequent access to health checks and treatments, which enable themselves to detect and address potential health problems timely, thereby reducing the risk of death. Additionally, sample size, duration of follow-up, and confounding factors associated with chronic diseases may account for different results [28]. To explore the deep-seated principles and reveal hidden confounding variables, in-depth scientific research is needed to enhance the accuracy and scope of the application of our results.

Excessive visceral adipose tissue may reduce the risk of mortality in older individuals via various mechanisms. First, previous studies have indicated that morbid obesity increased mortality risk; however, overweight or mild obesity reduced the risk of death in older individuals [29]. Most participants in our study were overweight or mildly obese. The older individuals with obesity in our study did not show classic markers of cardiometabolic risk such as high blood pressure, high TG levels, low HDL-C levels, high blood sugar, insulin resistance, or systemic inflammation. They were defined as healthy individuals with obesity and generally less possibility to suffer from adverse events [30]. In addition, visceral fat plays a key role as a cushion to protect vital organs, not only providing support, but also reducing the possibility of injury. Abdominal fat accumulation may mitigate the effects of this condition, reducing the likelihood of mortality [31]. Furthermore, fat around the viscera not only plays a role in energy storage, but also promotes the formation of adipose-derived stem cells (cells with regenerative potential). These cells are essential for the repair and regeneration of damaged tissues, play a positive role in improving health, and are expected to reduce mortality in the older population [32, 33]. Finally, although obesity is often associated with health threats of ascension in young individuals, older individuals may exhibit better health. Therefore, the effect of lifestyle habits on mortality cannot be ignored. Studies have shown that older adults who experience weight gain may have better eating habits, higher physical activity levels, more frequent social interactions, and better overall health. Increasing the dynamic activity of older individuals helps strengthen cardiorespiratory health, which in turn may weaken the positive association between obesity and poor health and ultimately improve overall survival rates [34–36]. However, the mechanism of action of adipose tissue in older populations and its possible protective function require further study. Exploring the interaction among obesity, metabolic status, and mortality risk in older individuals is a complex and evolving topic. Further exploration is necessary to reveal the underlying causes of these linkages, formulate plans to promote healthy aging, and optimize quality of life in older individuals.

Our study has several strengths. First, this was a prospective cohort study that explored the causal correlation between CVAI and the risk of mortality in older individuals and provided more advanced evidence than cross-sectional or retrospective studies. Second, our data were acquired from the CLHLS database, which contained comprehensive information and effectively promoteed research on older populations. Furthermore, sensitivity analysis was performed in our research, which improved the reliability of the results and confirmed that the association between CVAI and mortality risk was not only affected by short-term effects. Finally, we gained insights into potential effect modifiers through subgroup analysis, which highlighted the differences in the impact of CVAI on mortality risk in specific groups.

Some of the limitations of our study were as follows: First, the primary objective of this study was to assess the overall mortality risk without examining the specific mortality risk associated with particular diseases. Secondly, despite being structured as a prospective cohort study, some unmeasured confounding factors, such as social support, occupation, and family status, may exist that could affect the relationship between CVAI and death. Furthermore, our study was conducted only in a specific group of the older Chinese population, and this limitation may weaken the generalizability of its conclusions to other groups of different races or cultures. Additionally, the covariates involved in our study were mostly acquired by self-reports using a scientifically designed questionnaire, which may generate bias in data collection stage. Finally, the direct deletion method was used to handle missing data during data processing, which may have resulted in differences in conclusions.

In summary, our study strengthens the relevance of the CVAI and all-cause mortality, suggesting that the CVAI has significant utility in predicting all-cause mortality in the older population. The implementation of the CVAI in the older population as part of routine health assessment can help refine risk classification and encourage the implementation of targeted intervention strategies to optimize their health status. Further research is required to clarify the association between CVAI and mortality risk in older individuals.

## Conclusion

This prospective cohort study explored the association between CVAI and mortality in the older population using the CLHLS database. Notably, CVAI was linearly associated with all-cause mortality. Furthermore, a positive correlation was observed between higher CVAI levels and lower mortality, implying that the CVAI played a guardian role, especially in the age group of 65–79 years and in women. This discovery highlights that among young-older adults and women, managing and optimizing CVAI levels could potentially contribute to more effectively assessing and managing health risks, thereby reducing mortality rates and enhancing the quality of life for the older population.

## Data Availability

No datasets were generated or analysed during the current study.
